# Population bias in somatic measurement of microsatellite instability status

**DOI:** 10.1002/cam4.3294

**Published:** 2020-07-09

**Authors:** Michelle Saul, Kelsey Poorman, Hongseok Tae, Ari Vanderwalde, Phillip Stafford, David Spetzler, Wolfgang M. Korn, Zoran Gatalica, Jeff Swensen

**Affiliations:** ^1^ Caris Life Sciences Phoenix AZ USA; ^2^ The University of Tennessee Health Science Center and West Cancer Center Memphis TN USA

**Keywords:** checkpoint inhibitor, DNA mismatch repair, immunotherapy, microsatellite instability, next‐generation sequencing, population bias, reference genome, precision medicine

## Abstract

Microsatellite instability (MSI) is a key secondary effect of a defective DNA mismatch repair mechanism resulting in incorrectly replicated microsatellites in many malignant tumors. Historically, MSI detection has been performed by fragment analysis (FA) on a panel of representative genomic markers. More recently, using next‐generation sequencing (NGS) to analyze thousands of microsatellites has been shown to improve the robustness and sensitivity of MSI detection. However, NGS‐based MSI tests can be prone to population biases if NGS results are aligned to a reference genome instead of patient‐matched normal tissue. We observed an increased rate of false positives in patients of African ancestry with an NGS‐based diagnostic for MSI status utilizing 7317 microsatellite loci. We then minimized this bias by training a modified calling model that utilized 2011 microsatellite loci. With these adjustments 100% (95% CI: 89.1% to 100%) of African ancestry patients in an independent validation test were called correctly using the updated model. This poses not only a significant technical improvement but also has an important clinical impact on directing immune checkpoint inhibitor therapy.

## Graphical abstract

This work presents observations of a significant population bias in an NGS‐based MSI test and minimizes this bias using a computational‐based approach. The research poses not only a significant technical improvement to the NGS‐based MSI diagnostic, but has an important clinical impact on directing immune‐check point inhibitor therapy for late‐stage cancer patients of non‐European descent.

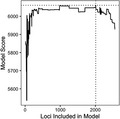

## 1 INTRODUCTION

Mismatch repair (MMR) proteins are responsible for recognizing and correcting insertion, deletion, and substitution mutations in DNA that can occur during replication, recombination, or from DNA damage. Mutations of MMR genes can lead to deficient mismatch repair (dMMR), which is frequently associated with loss of MMR protein expression. Microsatellite instability (MSI) is characterized by genome‐wide accumulation of insertion/deletion mutations in short repetitive DNA sequences, called microsatellites, resulting from dMMR mechanisms. Tumors with high levels of MSI (MSI‐H) can express an increased number of neoantigen peptides that signal a cell is cancerous and may increase the likelihood of response to immunotherapies.^1^ Clinical trials for the immune checkpoint inhibitor pembrolizumab have shown that MSI‐H status correlates with clinical response, agnostic of primary cancer site, leading to the first pan‐cancer drug approval by United States Food and Drug Administration's (FDA) for use of pembrolizumab in MSI‐H or dMMR patients.^2^

While no FDA approved companion diagnostic for MSI assessment is currently indicated to direct the use of pembrolizumab or other immune checkpoint inhibitors, the most common historical method for MSI classification in tumor specimens has been a polymerase chain reaction (PCR)‐based fragment analysis (FA) assay. FA classifies MSI status as high (MSI‐H), low (MSI‐L), or stable (MSS) by assessing DNA fragment length variation between tumor and patient‐matched normal tissue at five genomic loci (BAT25, BAT26, D2S123, D5S346, and D17S250), according to a 1997 National Cancer Institute (NCI) consensus meeting.^3^

Detection of MMR status by immunohistochemical (IHC) evaluation for the expression of MMR proteins MLH1, MSH2, MSH6, and PMS2 is an alternative method of determining patient eligibility for immune checkpoint inhibitors.^4^, ^5^, ^6^ Lack of expression in any one of these proteins indicates a dMMR system, which is strongly correlated with MSI‐H status. In most cases, detection of expression across all proteins indicates a proficient MMR (pMMR) system, which is strongly correlated with MSS status.

Recently, next‐generation sequencing (NGS) assays that assess thousands of microsatellites for MSI in solid tumor tissues have been developed that could improve robustness and sensitivity of MSI detection.^7^ Additionally, some NGS‐based methods do not require patient‐matched normal tissue which eliminates the need for collection and processing of normal tissue. This increases clinical access to MSI screening, as normal tissue is often not available from routine diagnostic biopsies.

In lieu of matched tissue, Vanderwalde, et al^7^ developed an NGS assay that aligns tumor genomic sequences to the human reference genome version hg19^8^ to assess variations between tumor and the reference genome at 7317 microsatellite loci. When aligning NGS results to a single reference genome, however, population‐based genomic bias is of concern. The initial human reference genome was derived from a limited (fewer than 20) number of individuals,^9^ and representation of normal variants was heavily biased toward populations of European ancestry.^10^ Findings from the 1000 Genomes Project indicate that variants can be specific to ancestral lines, and, in particular, individuals of African ancestry have more normal germline variations relative to other ancestral lines.^11^, ^12^ Benign, ancestral‐specific germline variants within microsatellite loci, not represented in the reference genome, can falsely appear as microsatellite insertion/deletion mutations. This can lead to an increased false detection of MSI in patients with these variants, and in particular, within patients of African ancestry.

In this study, we hypothesized that if an NGS method aligns potentially biased loci to a reference genome, then tumors with IHC pMMR status would have a significantly higher rate of false MSI detection when measured by NGS than when measured by FA in specimen with sequence variants known to be associated with African populations in the Genome Aggregation Database (gnomAD).^13^ To minimize this hypothesized bias, we modified the NGS MSI calling model described by Vanderwalde et al^7^ using an in‐silico feature selection analysis to select a potentially less biased set of microsatellite loci from the original set of 7317.

## 2 MATERIALS AND METHODS

All analyses were performed on deidentified, retrospective cases. As such, this research was covered under IRB Exemption, reviewed and determined by the Western Institutional Review Board (WIRB).

### 2.1 Data generation

#### 2.1.1 Sample collection

Formalin‐fixed paraffin‐embedded (FFPE) tissue samples from solid tumors across multiple cancer types were submitted over a 4‐year time period to a commercial CLIA‐certified laboratory (Caris Life Sciences) for genetic profiling as part of routine clinical care.

#### 2.1.2 Next‐generation Sequencing (NGS)

The details of NGS data generation have been previously described.^7^ Briefly, NGS was performed on genomic DNA isolated from FFPE samples using the NextSeq platform (Illumina, Inc). Prior to molecular testing, tumor enrichment was achieved by harvesting targeted tissue using manual microdissection techniques. A custom‐designed SureSelect XT assay was used to capture 592 whole‐gene targets (Agilent Technologies). All variants were detected with >99% confidence based on allele frequency and amplicon coverage, with an average sequencing depth of 750× and an analytic sensitivity of 5%. Sequencing alignment was compared with the reference genome hg19 from the UCSC Genome Browser database.

#### 2.1.3 Microsatellite identification

The details of microsatellite identification have been previously described.^7^ Briefly, 7317 microsatellite loci were identified by scanning short tandem repeats equal to or longer than five repeats of monomers (N = 6960), five repeats of dimers (N = 47), four repeats of trimers (N = 228), three repeats of tetramers (N = 57), or three repeats of pentamers (N = 25) from the panel target regions on the hg19 reference genome.

#### 2.1.4 Fragment analysis

MSI‐FA utilized a commercially available fluorescent multiplex PCR‐based method, MSI Analysis System (Promega Life Sciences). The system utilizes comparative analysis between enriched tumor tissue sample and nontumor (normal) tissue. Prior to molecular testing, tumor and matched normal tissue were collected by harvesting targeted tissue using manual microdissection techniques. Allelic profiles were generated for BAT‐25, BAT‐26, MONO‐27, NR‐21, and NR‐24 and compared between tumor and normal samples by a board‐certified clinical molecular geneticist.

#### 2.1.5 Immunohistochemistry

IHC analysis of mismatch repair proteins MSH6, MSH2, MLH1, and PMS2 was performed on full slides of FFPE tumor specimens using automated staining techniques on the Benchmark XT (Ventana) utilizing antibody clones MLH1 (M1), MSH2 (G219‐1129), MSH6 (44), and PMS2 (EPR3947). MMR status was determined to be deficient if staining indicated a complete loss of protein in any one of the four biomarkers in tumor cells and proficient if staining was present for all four proteins in tumor.

### 2.2 Study cohorts

#### 2.2.1 Historical cohort

A total of 6198 retrospective samples submitted to Caris Life Sciences for genetic profiling over a 4‐year time period, that had both FA MSI and NGS MSI results, were included in the historical cohort.

#### 2.2.2 Flagged cohort

Both a board‐certified clinical molecular geneticist and a board‐certified pathologist reviewed all samples submitted to Caris Life Sciences where IHC MMR and NGS MSI results were discordant. Samples with IHC pMMR results and NGS MSI‐H or NGS MSI‐equivocal results, which harbored sequence variants associated with African populations in gnomAD^13^ were flagged as having potential population‐biased NGS MSI results. All discordant samples with sufficient remnant material were tested by FA to determine MSI status. Over a 6‐month time period, a total of 64 flagged samples were tested for MSI by FA and included in the flagged cohort.

Genetic signatures of the flagged cohort were summarized utilizing population frequency data from gnomAD and compared to the genetic signatures of the historical cohort. To summarize the genetic signature of a sample, all alleles detected for that sample with reference SNP IDs (RSids) in gnomAD were identified. Population frequencies of each allele were collected from gnomAD via Bioconductor^14^ for the seven populations represented in the database (African/African‐American, Latino/Admixed American, Ashkenazi Jewish, East Asian, Finnish, Non‐Finnish European, and Other). An allele was then defined as supporting the population with the highest frequency of that allele. In the event of ties (eg, an allele had equivalent frequencies in two or more populations), the allele was defined as supporting all populations with the highest frequencies. For each sample, the number of alleles detected that supported each of the seven populations were totaled and the percentages of alleles supporting each population were calculated.

#### 2.2.3 Training and validation cohorts

The 6262 available samples from the flagged and historical cohorts were divided into training and independent validation cohorts. The validation cohort included 122 samples: 90 randomly selected historical samples equally distributed across FA MSI results (30 FA MSI‐H; 30 FA MSI‐L; 30 FA MSS) and 32 randomly selected flagged samples. The remaining 6140 samples were included in the training cohort. The 32 flagged samples included in training were used to assess how the model would perform on the flagged samples in the independent validation.

### 2.3 Bias assessment

Flagged cohort samples were used to assess bias. We hypothesized that, within the flagged cohort, FA results would agree more often with IHC MMR results than with NGS MSI results if NGS results were affected by population bias. A one‐sided exact test was performed to test the hypothesis that, in a cohort with discordant NGS MSI and IHC MMR results, the probability of FA results that agreed with IHC would be greater than 50%.

### 2.4 Model development

**FIGURE 1:**
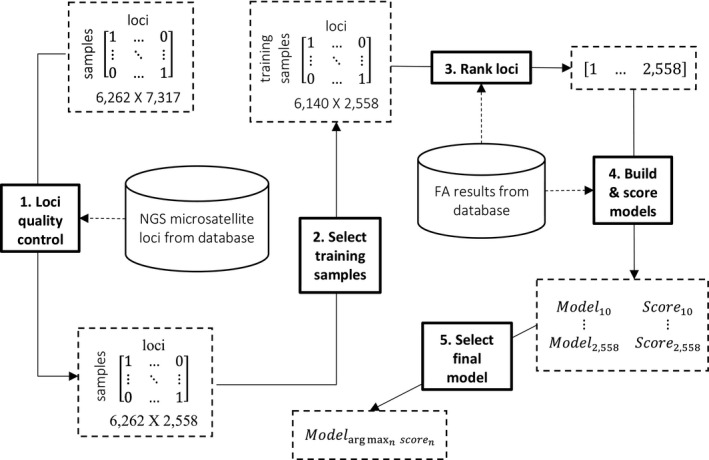
Diagram of model development process

An overview of the model development process is shown in Figure 1.

#### 2.4.1 Loci quality control

The quality of the original 7317 loci was assessed on all samples prior to model training. To be included in training, a locus had to meet the following minimum quality requirements:

1. Have a variant detected in at least one of the 6262 study samples.
2. Show repeatability when measured on replicates of the same sample.
3. Have an average sequencing coverage of at least 200×.

A total of 4759 loci were excluded during preprocessing. Of these, 4588 failed criterion 1, 10 failed criterion 2, and 161 failed criterion 3. The remaining 2558 loci were included in subsequent analyses.

#### 2.4.2 Loci ranking

The 2558 loci that met minimum quality requirements were ranked based on their ability to predict FA MSI status in the training samples. An informative locus was expected to have a higher number of variants detected in the FA MSI‐H samples than in the FA MSS samples. Analysis of variance (ANOVA) *P*‐values were computed to measure how informative a locus was by comparing the difference in the mean number of variants detected between the FA MSI‐H and FA MSS groups. If the mean of the FA MSS samples was higher than the mean of the FA MSI‐H samples, the *P*‐value for that locus was set to 1. Loci were ranked from lowest to highest *P*‐value.

#### 2.4.3 Calling models

The NGS MSI calling model classifies MSI status of a sample by totaling the number of variants detected across all microsatellite loci and comparing the total to predetermined thresholds. Totals greater than the upper threshold are classified as MSI‐H; totals less than the lower threshold are classified as MSS; and totals within the inclusive range of the two thresholds are classified as MSI equivocal. A total of 2549 calling models were built using between 10 and 2558 loci included sequentially by ranking.

#### 2.4.4 Thresholding

Thresholds for each model were calibrated on training data to optimize the separation between FA MSS and FA MSI‐H results. Samples with FA MSI‐L results were excluded from thresholding due to the fact that FA is subject to interpretation and different interpreters may classify FA MSI‐L calls as MSI‐H or MSS.

The first threshold was chosen to optimize the sum of sensitivity and specificity, and was calculated using methods provided in the pROC package^15^ for R version 3.5.2.^16^ The second threshold was chosen to ensure a maximum 3% false negative (FN) rate in the training population. It was calculated as the minimum of:

1. The maximum number of variants detected in FA MSS samples and
2. The third percentile of variants detected in the FA MSI‐H samples.

If the first threshold already met the 3% criterion, the second threshold had the effect of limiting the false positive (FP) calls.

#### 2.4.5 Model scoring

To score NGS models, NGS results were benchmarked against FA results. Results indicating eligibility for treatment by immune checkpoint inhibitors (FA MSI‐H and NGS MSI‐H) were considered positive, while results indicating ineligibility for immune checkpoint inhibitor treatment (FA MSI‐L, FA MSS, and NGS MSS) were considered negative. Model scores were calculated as true positive (TP) + true negative (TN) − false positive (FP) − false negative (FN). NGS equivocal calls did not contribute to model scores because operations protocol in a clinical setting for an NGS equivocal result often dictated that the sample be retested by FA to definitively determine MSI status. The number of loci corresponding to the maximum model score was selected as the optimal number of loci to include in the final model.

### 2.5 Model validation

Performance of the modified model was assessed initially via cross‐validation, to ensure the model was not overfitting the training data, and subsequently on the independent validation cohort, to confirm population bias was eliminated and overall performance was not hindered.

#### 2.5.1 Performance measures

Cross‐validation and independent validation performances of the modified NGS‐based calling model were assessed by calculating sensitivity, specificity, positive predictive value (PPV), and negative predictive value (NPV) benchmarked against FA MSI results. For these calculations, results that indicated eligibility for treatment by immune checkpoint inhibitors (FA MSI‐H and NGS MSI‐H) were considered positive, while results that were not eligible for treatment by immune checkpoint inhibitors (FA MSI‐L, FA MSS, and NGS MSS) were considered negative. Again, NGS MSI‐equivocal results were excluded from analysis as the clinical protocol for NGS MSI‐equivocal results often dictated that samples be tested with FA. Percent equivocal values were reported for reference along with performance measures. Due to the fact that the historical validation cohort was enriched for FA MSI‐L and FA MSS samples, performance measures on this cohort were evaluated on the enriched population as well as a prevalence adjusted population. Prevalence values for MSI‐H, MSI‐L, and MSS used for adjustment were estimated from the FA MSI results in the training cohort.

#### 2.5.2 Cross‐validation

Ten iterations of threefold cross‐validation were performed on the training data to estimate model utility on unknown cases. Data were randomized to folds preserving distributions of FA MSI calls using the Caret package^17^ in R version 3.5.2. Training folds were used to rank loci and build and score models. The optimal model was then applied to the testing fold and performance measures were computed. Threefold CV was repeated for 10 different randomization seeds, and performance measures were averaged over these 10 runs.

### 2.6 Reference genome version comparison

The original NGS MSI calling model used human reference genome hg19 for alignment, and is the reference genome version utilized for this study. However, a newer version of the human reference genome, hg38, has since been released. Effects of aligning to this newer version were assessed on the independent validation cohort. MSI loci variants were recomputed for all 7317 microsatellite loci after alignment to hg38 and compared to the number of variants detected under hg19 alignment.

## 3 RESULTS

### 3.1 Bias assessment

All 64 samples in the flagged cohort had FA MSS results. The observed probability that the FA MSI results were concordant with the pMMR IHC results in this cohort was 100% (95% CI: 95%, 100%) and was statistically significantly greater than the probability expected by chance (*P* « .001), suggesting a strong bias in the NGS results. The performance measures of the NGS results on the flagged cohort are presented in Table 1.

**TABLE 1:** Original NGS model performance on flagged cohort samples

	IHC Result	FA Result	Original NGS Model Results (N)			Specificity % (95% CI^a^)	Equivocal % (95% CI^a^)
			MSI‐H	Equivocal	MSS		
Flagged Cohort	pMMR	MSS	16	48	0	0 (0.0, 20.6)	75 (62.6, 85.0)

^a^ Confidence intervals (CI) calculated by Clopper‐Pearson method.

### 3.2 Demographics

Gender and cancer types were compared across cohorts stratified by FA MSI status using chi‐square test. No significant differences were found. Age was compared across cohorts stratified by FA MSI status using analysis of variance (ANOVA). The flagged cohorts had slightly lower average ages than the historical cohorts, which supports observations of lower ages of cancer in African‐American populations.^18^ Available demographics by FA MSI status for historical training, historical validation, flagged training, and flagged validation cohorts are shown in Table S1.

Ancestry was summarized as described in the methods section. On average, 43% of the alleles for flagged cohort samples supported African/African‐American population, with a minimum of 38%, while the historical cohort averaged 20%, suggesting the flagged cohort samples had genetic signatures more consistent with African/African‐American ancestry than the average sample from the historical cohort. A summary of the number of alleles supporting the seven populations in gnomAD for each of the flagged samples can be found in Table S2. Additionally, a comparison of the distribution of alleles by population between the historical cohort and the flagged cohort can be found in Figure S1.

### 3.3 Validation results

Cross‐validation and independent validation results are summarized in Table 2. Most notably, results showed a drastic improvement of specificity in the flagged cohorts. Specificity on the flagged cohort went from 0% with the original model to 94% in the cross‐validated models within the training set, and from 0% with the original model to 100% in the final modified model during validation. Additionally, PPV had a moderate increase from 93.2% in the original model to 96.4% in the cross‐validated models.

**TABLE 2:** Performance of NGS model methods on training and validation cohorts

NGS MSI Method	Cohort	FA Result	NGS Results (N)			Sensitivity % (95% CI^a^)	Specificity % (95% CI^a^)	PPV % (95% CI^a^)	NPV % (95% CI^a^)	Equivocal % (95% CI^a^)
			MSI‐H	Equivocal	MSS					
Original Model	Historical Training	MSI‐H	399	7	16	96.1 (93.8, 97.8)	99.5 (99.3, 99.7)	93.2 (90.4, 95.4)	99.7 (99.5, 99.8)	1.0 (0.8, 1.3)
		MSI‐L	3	5	27					
		MSS	26	52	5573					
	Flagged Training	MSS	9	23	0	—	0.0 (0, 33.6)	—	—	71.9 (53.3, 86.3)
Modified Models Averaged over 10 X threefold CV	Historical Training	MSI‐H	405	7.9	9.1	97.8 (95.9, 99.0)	99.7 (99.6, 99.9)	96.4 (94.2, 98.0)	99.8 (99.7, 99.9)	0.3 (0.2, 0.5)
		MSI‐L	3.7	2.1	29.2					
		MSS	11.3	8.8	5630.9					
	Flagged Training	MSS	0.2	1.1	30.7	—	99.4 (88.8, 100)	—	—	3.4 (0.1, 16.2)
Original Model	Historical Validation	MSI‐H	28	1	1	96.6 (82.2, 99.9)	86.0 (74.2, 93.7)	77.8 (60.8, 89.9)	98.0 (89.4, 99.9)	4.4 (1.2, 11)
		MSI‐L	8	3	19					
		MSS	0	0	30					
	Historical Validation (Adjusted)	MSI‐H	5.77	0.21	0.21	96.5 (54.1, 100)	99.8 (95.7, 100)	97.6 (54.1, 100)	99.7 (95.7, 100)	0.3 (0.0, 4.0)
		MSI‐L	0.14	0.05	0.32					
		MSS	0	0	83.3					
	Flagged Validation	MSS	7	25	0	—	0.0 (0.0, 41)	—	—	78.1 (60.0, 90.7)
Final Modified Model	Historical Validation	MSI‐H	29	1	0	100 (88.1, 100)	84.7 (73.0, 92.8)	76.3 (59.8, 88.6)	100 (92.9, 100)	2.2 (0.3, 7.8)
		MSI‐L	9	1	20					
		MSS	0	0	30					
	Historical Validation (Adjusted)	MSI‐H	5.98	0.21	0	100 (54.1, 100)	99.8 (95.7, 100)	97.6 (54.1, 100)	100 (95.7, 100)	0.3 (0, 4.0)
		MSI‐L	0.15	0.02	0.34					
		MSS	0	0	83.3					
	Flagged Validation	MSS	0	0	32	—	100 (89.1, 100)	—	—	0.0 (0.0, 10.9)

^a^ Confidence intervals (CI) calculated by Clopper‐Pearson method. All decimal values were rounded to the nearest whole number for CI calculations.

### 3.4 Final model selection

Because cross‐validation results were satisfactory, the methodology was used on the full set of training data to create a final modified NGS MSI calling model. The 2558 loci that passed quality control were included in the analysis. The optimal model score corresponded to the model containing 2011 loci, eliminating a total of 547 uninformative microsatellite loci. Additional figures illustrating model selection are provided in the supplementary materials. Final model results on the Flagged cohort are shown in Table 3. For the interested reader, results of the final model compared to the original model are shown by cancer type in Table S3.

**TABLE 3:** Updated NGS model performance on flagged cohort samples

	IHC Result	FA Result	Updated NGS Model Results (N)			Specificity % (95% CI^a^)	Equivocal % (95% CI^a^)
			MSI‐H	Equivocal	MSS		
Flagged Cohort	pMMR	MSS	0	0	64	100 (94.4, 100)	0 (0, 5.6)

^a^ Confidence intervals (CI) calculated by Clopper‐Pearson method.

### 3.5 Reference genome version comparison

Overall 892 674 variants were assessed (7317 loci across 122 samples). Of these, only two variants were discordant between the hg19 and hg38 alignments. One microsatellite variant was detected under hg19 alignment, but not detected under hg38 alignment due to different alignments on the reverse strand in hg38. The other microsatellite variant was detected under hg38 alignment but not under hg19 alignment, due to low variant allele frequency.

The discrepant variant calls were detected in two samples. However, discrepancies did not affect the final MSI status determination. Both samples were classified as MSI‐H under both the hg19 and hg38 alignments. Therefore, there was 100% concordance of NGS MSI calls between the genome versions. This result suggests that all conclusions drawn in the analyses of hg19‐aligned samples are valid in samples aligned to hg38.

## 4 DISCUSSION

In this study, a bias was observed in NGS MSI test results within a cohort of patients with presumed African ancestry. Our goal was to refine the selection of microsatellite loci used to determine MSI status and improve the specificity of the test for patients prone to the observed bias. We accomplished this by training a new model using a large database of pan‐cancer sequencing data, including flagged patients who previously exhibited a false positive NGS‐based MSI test result compared to FA or IHC.

Initial refinement required a quality assessment of the 7317 original loci, which eliminated over half the loci. Next, a model‐building process eliminated noninformative loci resulting in a final set of 2011 loci that enhanced overall performance of the calling model. In particular, the updated model increased specificity in flagged cases by 100% in an independent validation, and increased overall PPV by approximately 3% in a cross‐validation assessment. Overall specificity was not affected due to the large number of MSS samples in the general population. However, the 3% increase in PPV seen in cross‐validation results suggests roughly 3% of MSI‐H samples in the general population were prone to the bias observed in this study.

Performance of MSI diagnostics are important to direct the use of immunotherapies due to the immunological response induced in patients whose tumors produce large numbers of neoantigens. This immunological landscape is not present in MSS patients, so false positive results may lead to patients receiving a pharmacological agent unlikely to have clinical benefit.

While the performance of FA MSI diagnostics have been well established, NGS‐based methods for MSI assessment offer several advantages: they can extend the availability of MSI diagnostics to patients whose tumor biopsies do not have sufficient normal tissue for MSI assessment by FA; they can potentially provide a more accurate assessment of genomic signatures due to the large number of microsatellite loci surveyed; and they can be assessed as part of comprehensive genetic profiling panels, resulting in conservation of tissue and reduced time‐to‐results, allowing clinicians to select appropriate therapies more quickly.

The work presented here suggests that NGS‐based tests that are affected by population biases can significantly benefit from training models using data from unbiased methods. Although this work focused on MSI, similar approaches could likely be utilized to minimize biases in NGS‐based tests for other biomarkers as well.

## DISCLOSURES

MS, KP, HT, PS, DS, WMK, ZG, and JS are employees of Caris Life Sciences.

## AUTHOR CONTRIBUTIONS

KP and JS conceived of the presented idea. MS developed the theory and performed the computations. HT provided data. ZG and JS encouraged MS to investigate population signatures and supervised the findings of this work. All authors discussed the results and contributed to the final manuscript.

## Supporting information

Supplementary Material

Supplementary Material

## Data Availability

The authors confirm that the data supporting the findings of this study are available within the article's supplementary materials.
