# Supplementary material for: Population bias in somatic measurement of microsatellite instability status
Source: Cancer Med. 2020 Jul 9;9(17):6452–60. doi: 10.1002/cam4.3294 (PMC7476819; doi:10.1002/cam4.3294)
Supplement: Supplementary file 1 — Supplementary Material [file CAM4-9-6452-s001.docx]

| Supplemental Table 1: Cohort demographics by FA MSI status | | | | | |
| --- | --- | --- | --- | --- | --- |
|  | Historical Training | Flagged Training | Historical Validation | Flagged Validation |  |
|  | (N = 6,108) | (N = 32) | (N = 90) | (N = 32) |  |
| **FA MSI-H** | N = 422 | N = 0 | N = 30 | N = 0 |  |
| Age |  |  |  |  | 0.61 |
| Mean | 65 | --- | 65 | --- |  |
| Median | 66 | --- | 70 | --- |  |
| Range | 22-90 | --- | 25-90 | --- |  |
| Gender |  |  |  |  | 0.93 |
| Female | 334 (79.1%) | --- | 23 (76.7%) | --- |  |
| Male | 88 (20.9%) | --- | 7 (23.3%) | --- |  |
| Cancer Type |  |  |  |  | 0.88 |
| Breast | 2 (0.5%) | --- | 0 (0%) | --- |  |
| Gastrointestinal | 185 (43.8%) | --- | 12 (40%) | --- |  |
| Genitourinary | 3 (0.7%) | --- | 0 (0%) | --- |  |
| Gynecologic | 220 (52.1%) | --- | 18 (60%) | --- |  |
| Male Genital Tract | 1 (0.2%) | --- | 0 (0%) | --- |  |
| Other | 7 (1.7%) | --- | 0 (0%) | --- |  |
| Thoracic | 4 (0.9%) | --- | 0 (0%) | --- |  |
| **FA MSI-L** | N = 35 | N = 0 | N = 30 | N = 0 |  |
| Age |  |  |  |  | 0.94 |
| Mean | 61 | --- | 64 | --- |  |
| Median | 65 | --- | 64 | --- |  |
| Range | 31-79 | --- | 44-82 | --- |  |
| Gender |  |  |  |  | 0.73 |
| Female | 22 (62.9%) | --- | 21 (70%) | --- |  |
| Male | 13 (37.1%) | --- | 9 (30%) | --- |  |
| Cancer Type |  |  |  |  | 0.4 |
| Gastrointestinal | 18 (51.4%) | --- | 11 (36.7%) | --- |  |
| Gynecologic | 14 (40%) | --- | 17 (56.7%) | --- |  |
| Other | 2 (5.7%) | --- | 1 (3.3%) | --- |  |
| Sarcoma | 1 (2.9%) | --- | 0 (0%) | --- |  |
| Skin | 0 (0%) | --- | 1 (3.3%) | --- |  |
| **FA MSS** | N = 5,651 | N = 32 | N = 30 | N = 32 |  |
| Age |  |  |  |  | 0.02 |
| Mean | 66 | 62 | 65 | 60 |  |
| Median | 67 | 65 | 66 | 60 |  |
| Range | 7-90 | 38-83 | 42-93 | 28-82 |  |
| Gender |  |  |  |  | 0.69 |
| Female | 3,539 (62.6%) | 23 (71.9%) | 20 (66.7%) | 21 (65.6%) |  |
| Male | 2,112 (37.4%) | 9 (28.1%) | 10 (33.3%) | 11 (34.4%) |  |
| Cancer Type |  |  |  |  | 0.27 |
| Breast | 175 (3.1%) | 0 (0%) | 0 (0%) | 2 (6.2%) |  |
| Gastrointestinal | 2621 (46.4%) | 15 (46.9%) | 14 (46.7%) | 9 (28.1%) |  |
| Genitourinary | 173 (3.1%) | 2 (6.2%) | 1 (3.3%) | 3 (9.4%) |  |
| Gynecologic | 1640 (29%) | 8 (25%) | 8 (26.7%) | 10 (31.2%) |  |
| Lymphoma | 4 (0.1%) | 0 (0%) | 0 (0%) | 0 (0%) |  |
| Male Genital Tract | 1 (0%) | 0 (0%) | 0 (0%) | 0 (0%) |  |
| Neuroendocrine | 77 (1.4%) | 2 (6.2%) | 0 (0%) | 1 (3.1%) |  |
| Other | 169 (3%) | 2 (6.2%) | 3 (10%) | 2 (6.2%) |  |
| Brain | 20 (0.4%) | 0 (0%) | 0 (0%) | 0 (0%) |  |
| Sarcoma | 62 (1.1%) | 0 (0%) | 0 (0%) | 1 (3.1%) |  |
| Skin | 125 (2.2%) | 0 (0%) | 1 (3.3%) | 0 (0%) |  |
| Thoracic | 584 (10.3%) | 3 (9.4%) | 3 (10%) | 4 (12.5%) |  |

| Supplemental Table 2: Number of alleles supporting genetic signatures of ancestry* | | | | | | | |
| --- | --- | --- | --- | --- | --- | --- | --- |
| **Sample** | **African/ African-American** | **Latino/ Admixed American** | **Ashkenazi Jewish** | **East Asian** | **Finnish** | **Non-Finnish European** | **Other** |
| 1 | 356 | 87 | 94 | 113 | 84 | 48 | 40 |
| 2 | 326 | 85 | 93 | 109 | 79 | 41 | 33 |
| 3 | 327 | 95 | 93 | 96 | 83 | 51 | 37 |
| 4 | 355 | 81 | 89 | 98 | 84 | 49 | 39 |
| 5 | 373 | 90 | 82 | 97 | 77 | 49 | 42 |
| 6 | 353 | 89 | 101 | 114 | 97 | 54 | 43 |
| 7 | 353 | 85 | 82 | 102 | 75 | 41 | 34 |
| 8 | 359 | 88 | 86 | 108 | 72 | 43 | 37 |
| 9 | 301 | 88 | 94 | 107 | 81 | 45 | 37 |
| 10 | 370 | 92 | 80 | 101 | 81 | 42 | 39 |
| 11 | 345 | 90 | 108 | 91 | 96 | 48 | 37 |
| 12 | 295 | 83 | 98 | 102 | 90 | 50 | 36 |
| 13 | 358 | 93 | 86 | 107 | 81 | 53 | 38 |
| 14 | 355 | 103 | 96 | 107 | 86 | 48 | 38 |
| 15 | 370 | 82 | 84 | 106 | 73 | 50 | 40 |
| 16 | 338 | 97 | 92 | 108 | 87 | 53 | 39 |
| 17 | 396 | 80 | 80 | 108 | 70 | 39 | 38 |
| 18 | 362 | 89 | 84 | 110 | 79 | 49 | 35 |
| 19 | 368 | 82 | 91 | 99 | 73 | 52 | 43 |
| 20 | 376 | 83 | 102 | 109 | 79 | 46 | 37 |
| 21 | 357 | 81 | 93 | 104 | 85 | 48 | 37 |
| 22 | 364 | 81 | 81 | 108 | 77 | 37 | 34 |
| 23 | 340 | 98 | 94 | 119 | 86 | 54 | 43 |
| 24 | 374 | 78 | 95 | 107 | 65 | 38 | 36 |
| 25 | 364 | 89 | 91 | 98 | 74 | 56 | 33 |
| 26 | 332 | 89 | 97 | 111 | 81 | 56 | 40 |
| 27 | 351 | 85 | 82 | 93 | 76 | 38 | 41 |
| 28 | 348 | 93 | 91 | 105 | 81 | 48 | 42 |
| 29 | 349 | 72 | 106 | 88 | 99 | 57 | 40 |
| 30 | 359 | 84 | 96 | 111 | 87 | 47 | 39 |
| 31 | 334 | 82 | 91 | 95 | 91 | 49 | 46 |
| 32 | 319 | 90 | 96 | 99 | 100 | 59 | 40 |
| 33 | 319 | 78 | 94 | 106 | 83 | 57 | 38 |
| 34 | 344 | 83 | 88 | 100 | 79 | 49 | 40 |
| 35 | 319 | 93 | 101 | 117 | 89 | 48 | 37 |
| 36 | 329 | 91 | 95 | 104 | 88 | 46 | 36 |
| 37 | 311 | 96 | 87 | 107 | 82 | 51 | 40 |
| 38 | 365 | 87 | 91 | 107 | 90 | 46 | 32 |
| 39 | 373 | 88 | 80 | 83 | 76 | 47 | 40 |
| 40 | 367 | 87 | 91 | 94 | 90 | 45 | 39 |
| 41 | 362 | 84 | 79 | 108 | 80 | 51 | 38 |
| 42 | 340 | 83 | 92 | 108 | 77 | 43 | 34 |
| 43 | 319 | 84 | 85 | 111 | 91 | 48 | 41 |
| 44 | 366 | 89 | 90 | 105 | 81 | 44 | 43 |
| 45 | 377 | 80 | 90 | 91 | 80 | 48 | 42 |
| 46 | 338 | 81 | 95 | 119 | 94 | 50 | 41 |
| 47 | 357 | 91 | 85 | 104 | 80 | 49 | 36 |
| 48 | 339 | 88 | 87 | 96 | 84 | 47 | 34 |
| 49 | 288 | 85 | 92 | 93 | 91 | 45 | 35 |
| 50 | 347 | 88 | 93 | 107 | 88 | 50 | 39 |
| 51 | 339 | 84 | 92 | 100 | 82 | 49 | 42 |
| 52 | 340 | 74 | 81 | 99 | 77 | 47 | 37 |
| 53 | 339 | 94 | 81 | 101 | 83 | 48 | 36 |
| 54 | 380 | 93 | 86 | 99 | 80 | 43 | 35 |
| 55 | 314 | 96 | 108 | 103 | 87 | 67 | 44 |
| 56 | 313 | 95 | 97 | 116 | 89 | 50 | 41 |
| 57 | 299 | 93 | 96 | 110 | 97 | 43 | 35 |
| 58 | 382 | 83 | 92 | 108 | 79 | 50 | 39 |
| 59 | 370 | 90 | 82 | 109 | 72 | 46 | 40 |
| 60 | 308 | 87 | 93 | 100 | 87 | 59 | 45 |
| 61 | 384 | 95 | 97 | 104 | 85 | 55 | 37 |
| 62 | 323 | 99 | 91 | 96 | 99 | 54 | 43 |
| 63 | 339 | 90 | 100 | 123 | 77 | 51 | 36 |
| 64 | 343 | 80 | 94 | 100 | 79 | 47 | 34 |
| *Alleles were detected for each sample using NGS sequencing. For alleles with reference SNP IDs in gnomAD, population frequencies were collected. An allele was defined as supporting the ancestry with the highest frequency of that allele. The numbers indicate the total number of alleles supporting each population per sample. | | | | | | | |

| \| **Supplementary Figure 1:** Sample population allele frequencies by cohort. \| \| \| \| --- \| --- \| --- \| \| 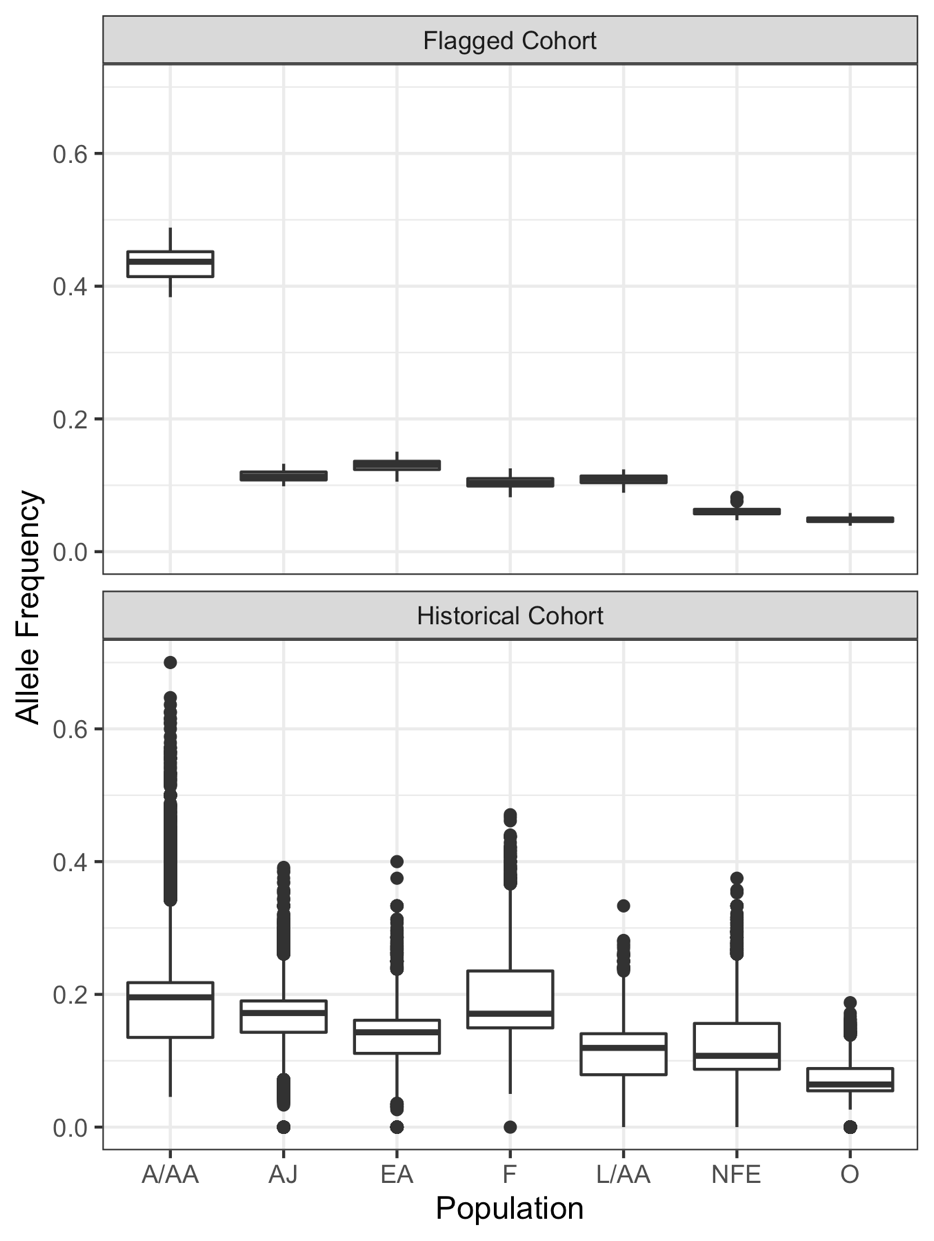 \| **Population Key** \| \| \| A/AA  AJ  EA  F  L/AA  NFE  O \| African/African-American  Ashkenazi Jewish  East Asian  Finnish  Latino/Admixed American  Non-Finnish European  Other \| \| Samples in the flagged cohort had significantly more African/African-American allele frequencies than any other population. By comparison, allele frequencies for samples in the historical cohort were more evenly distributed. \| \| \|   **Table 3:** Performance of NGS model methods by cancer type. | | | | | | | | | | |  |
| --- | --- | --- | --- | --- | --- | --- | --- | --- | --- | --- | --- | --- | --- | --- | --- | --- | --- | --- | --- | --- | --- | --- |
| **Cancer Type** | **NGS MSI Method** | **FA Result** | **NGS Results (N)** | | | **Sensitivity % (95% CI*)** | **Specificity % (95% CI*)** | **PPV %**  **(95% CI*)** | **NPV % (95% CI*)** | **Equivocal % (95% CI*)** | |
|  |  |  | **MSI-H** | **Equivocal** | **MSS** |  |  |  |  |  |  |
| Breast | Original | MSI-H | 2 | 0 | 0 | 100 (15.8, 100) | 98.8 (95.9, 99.9) | 50 (6.8, 93.2) | 100 (97.9, 100) | 2.8 (0.9, 6.4) | |
|  |  | MSI-L | 0 | 0 | 0 |  |  |  |  |  |  |
|  |  | MSS | 2 | 5 | 170 |  |  |  |  |  |  |
|  | Modified | MSI-H | 2 | 0 | 0 | 100 (15.8, 100) | 99.4 (96.9, 100) | 66.7 (9.4, 99.2) | 100 (97.9, 100) | 0.6 (0, 3.1) | |
|  |  | MSI-L | 0 | 0 | 0 |  |  |  |  |  |  |
|  |  | MSS | 1 | 1 | 175 |  |  |  |  |  |  |
| Gastro-intestinal | Original | MSI-H | 191 | 0 | 6 | 97 (93.5, 98.9) | 99.4 (99, 99.6) | 91.8 (87.2, 95.2) | 99.8 (99.5, 99.9) | 1.5 (1.1, 2) | |
|  |  | MSI-L | 0 | 3 | 26 |  |  |  |  |  |  |
|  |  | MSS | 17 | 39 | 2603 |  |  |  |  |  |  |
|  | Modified | MSI-H | 192 | 2 | 3 | 98.5 (95.6, 99.7) | 100 (99.9, 100) | 100 (98.1, 100) | 99.9 (99.7, 100) | 0.2 (0.1, 0.4) | |
|  |  | MSI-L | 0 | 2 | 27 |  |  |  |  |  |  |
|  |  | MSS | 0 | 1 | 2658 |  |  |  |  |  |  |
| Genito-urinary | Original | MSI-H | 3 | 0 | 0 | 100 (29.2, 100) | 98.3 (95, 99.6) | 50 (11.8, 88.2) | 100 (97.9, 100) | 2.7 (0.9, 6.3) | |
|  |  | MSI-L | 0 | 0 | 0 |  |  |  |  |  |  |
|  |  | MSS | 3 | 5 | 171 |  |  |  |  |  |  |
|  | Modified | MSI-H | 3 | 0 | 0 | 100 (29.2, 100) | 100 (98, 100) | 100 (29.2, 100) | 100 (98, 100) | 0 (0, 2) | |
|  |  | MSI-L | 0 | 0 | 0 |  |  |  |  |  |  |
|  |  | MSS | 0 | 0 | 179 |  |  |  |  |  |  |
| Gynecologic | Original | MSI-H | 221 | 8 | 9 | 96.1 (92.7, 98.2) | 99.1 (98.5, 99.5) | 93.6 (89.7, 96.4) | 99.4 (99, 99.7) | 1.9 (1.3, 2.6) | |
|  |  | MSI-L | 11 | 5 | 15 |  |  |  |  |  |  |
|  |  | MSS | 15 | 24 | 1627 |  |  |  |  |  |  |
|  | Modified | MSI-H | 230 | 6 | 2 | 99.1 (96.9, 99.9) | 99.7 (99.3, 99.9) | 97.9 (95.1, 99.3) | 99.9 (99.6, 100) | 0.7 (0.4, 1.2) | |
|  |  | MSI-L | 12 | 2 | 17 |  |  |  |  |  |  |
|  |  | MSS | 5 | 6 | 1655 |  |  |  |  |  |  |
| Lymphoma | Original | MSI-H | 0 | 0 | 0 | --- | 100 (39.8, 100) | --- | 100 (39.8, 100) | 0 (0, 60.2) | |
|  |  | MSI-L | 0 | 0 | 0 |  |  |  |  |  |  |
|  |  | MSS | 0 | 0 | 4 |  |  |  |  |  |  |
|  | Modified | MSI-H | 0 | 0 | 0 | --- | 100 (39.8, 100) | --- | 100 (39.8, 100) | 0 (0, 60.2) | |
|  |  | MSI-L | 0 | 0 | 0 |  |  |  |  |  |  |
|  |  | MSS | 0 | 0 | 4 |  |  |  |  |  |  |
| Male Genital Tract Malignancy | Original | MSI-H | 0 | 0 | 1 | 0 (0, 97.5) | 100 (2.5, 100) | --- | 50 (1.3, 98.7) | 0 (0, 84.2) | |
|  |  | MSI-L | 0 | 0 | 0 |  |  |  |  |  |  |
|  |  | MSS | 0 | 0 | 1 |  |  |  |  |  |  |
|  | Modified | MSI-H | 0 | 0 | 1 | 0 (0, 97.5) | 100 (2.5, 100) | --- | 50 (1.3, 98.7) | 0 (0, 84.2) | |
|  |  | MSI-L | 0 | 0 | 0 |  |  |  |  |  |  |
|  |  | MSS | 0 | 0 | 1 |  |  |  |  |  |  |
| Neuro-endocrine tumors | Original | MSI-H | 0 | 0 | 0 | --- | 98.7 (92.9, 100) | 0 (0, 97.5) | 100 (95.2, 100) | 5 (1.4, 12.3) | |
|  |  | MSI-L | 0 | 0 | 0 |  |  |  |  |  |  |
|  |  | MSS | 1 | 4 | 75 |  |  |  |  |  |  |
|  | Modified | MSI-H | 0 | 0 | 0 | --- | 100 (95.4, 100) | --- | 100 (95.4, 100) | 1.2 (0, 6.8) | |
|  |  | MSI-L | 0 | 0 | 0 |  |  |  |  |  |  |
|  |  | MSS | 0 | 1 | 79 |  |  |  |  |  |  |
| None of These Apply | Original | MSI-H | 6 | 0 | 1 | 85.7 (42.1, 99.6) | 100 (97.9, 100) | 100 (54.1, 100) | 99.4 (96.8, 100) | 3.2 (1.2, 6.9) | |
|  |  | MSI-L | 0 | 0 | 3 |  |  |  |  |  |  |
|  |  | MSS | 0 | 6 | 170 |  |  |  |  |  |  |
|  | Modified | MSI-H | 7 | 0 | 0 | 100 (59, 100) | 100 (97.9, 100) | 100 (59, 100) | 100 (97.9, 100) | 0 (0, 2) | |
|  |  | MSI-L | 0 | 0 | 3 |  |  |  |  |  |  |
|  |  | MSS | 0 | 0 | 176 |  |  |  |  |  |  |
| Primary Brain Tumors | Original | MSI-H | 0 | 0 | 0 | --- | 100 (81.5, 100) | --- | 100 (81.5, 100) | 10 (1.2, 31.7) | |
|  |  | MSI-L | 0 | 0 | 0 |  |  |  |  |  |  |
|  |  | MSS | 0 | 2 | 18 |  |  |  |  |  |  |
|  | Modified | MSI-H | 0 | 0 | 0 | --- | 95 (75.1, 99.9) | 0 (0, 97.5) | 100 (82.4, 100) | 0 (0, 16.8) | |
|  |  | MSI-L | 0 | 0 | 0 |  |  |  |  |  |  |
|  |  | MSS | 1 | 0 | 19 |  |  |  |  |  |  |
| Sarcoma | Original | MSI-H | 0 | 0 | 0 | --- | 100 (94.1, 100) | --- | 100 (94.1, 100) | 3.1 (0.4, 10.8) | |
|  |  | MSI-L | 0 | 0 | 1 |  |  |  |  |  |  |
|  |  | MSS | 0 | 2 | 61 |  |  |  |  |  |  |
|  | Modified | MSI-H | 0 | 0 | 0 | --- | 100 (94.3, 100) | --- | 100 (94.3, 100) | 0 (0, 5.6) | |
|  |  | MSI-L | 0 | 0 | 1 |  |  |  |  |  |  |
|  |  | MSS | 0 | 0 | 63 |  |  |  |  |  |  |
| Skin Cancer | Original | MSI-H | 0 | 0 | 0 | --- | 100 (97.1, 100) | --- | 100 (97.1, 100) | 0.8 (0, 4.3) | |
|  |  | MSI-L | 0 | 0 | 1 |  |  |  |  |  |  |
|  |  | MSS | 0 | 1 | 125 |  |  |  |  |  |  |
|  | Modified | MSI-H | 0 | 0 | 0 | --- | 100 (97.1, 100) | --- | 100 (97.1, 100) | 0.8 (0, 4.3) | |
|  |  | MSI-L | 0 | 0 | 1 |  |  |  |  |  |  |
|  |  | MSS | 0 | 1 | 125 |  |  |  |  |  |  |
| Thoracic | Original | MSI-H | 4 | 0 | 0 | 100 (39.8, 100) | 99.3 (98.2, 99.8) | 50 (15.7, 84.3) | 100 (99.4, 100) | 2 (1, 3.5) | |
|  |  | MSI-L | 0 | 0 | 0 |  |  |  |  |  |  |
|  |  | MSS | 4 | 12 | 578 |  |  |  |  |  |  |
|  | Modified | MSI-H | 4 | 0 | 0 | 100 (39.8, 100) | 100 (99.4, 100) | 100 (39.8, 100) | 100 (99.4, 100) | 0.2 (0, 0.9) | |
|  |  | MSI-L | 0 | 0 | 0 |  |  |  |  |  |  |
|  |  | MSS | 0 | 1 | 593 |  |  |  |  |  |  |

*Confidence intervals (CI) calculated by Clopper-Pearson method. All decimal values were rounded to the nearest whole number for CI calculations.

| **Supplementary Figure 2:** Number of loci included in model versus model score. The dotted line indicates the maximum score at 2011 Loci. |
| --- |
| 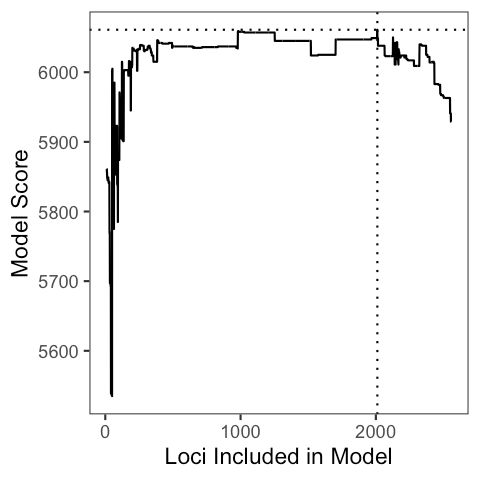 |

| **Supplementary Figure 3:** Depiction of the final model selected on the training data; thresholds shown as horizontal dotted lines. |
| --- |
| 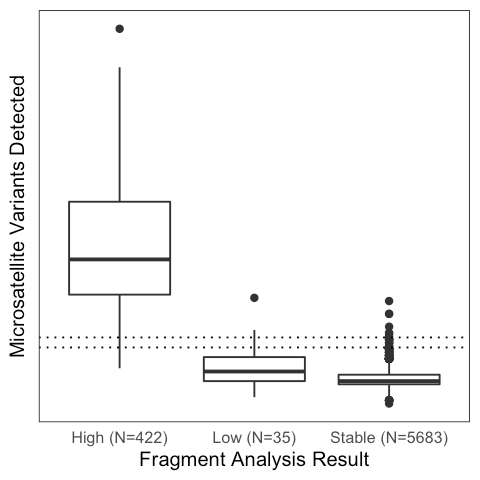 |
